# Evaluation of Real-world treatment patterns in Japanese patients with cGVHD: A retrospective claims database study

**DOI:** 10.1007/s12185-026-04158-6

**Published:** 2026-03-18

**Authors:** Junya Kanda, Kittima Wattanakamolkul, Hideyuki Muromine, Kaname Shiga

**Affiliations:** 1 Department of Hematology, Kyoto University Graduate School of Medical Sciences, Kyoto, Japan; 2 Integrated Market Access, Johnson & Johnson, 3-5-2, Nishi-Kanda, Chiyoda-Ku, Tokyo, 101-0065 Japan; 3 Communication and Public Affairs, Johnson & Johnson, Tokyo, Japan; 4 Medical Affairs, Johnson & Johnson, Tokyo, Japan

**Keywords:** cGVHD, Japan, Medical data vision database, Retrospective

## Abstract

This retrospective cohort study using the medical data vision (MDV) database included adult patients who had confirmed diagnosis of cGVHD between 2003 and 2023, were prescribed a steroid prior to diagnosis of cGVHD, and received mycophenolate mofetil (MMF), ibrutinib, or ruxolitinib as second- or later-line therapy. Duration of treatment (DoT) and steroid dose reduction during second-line therapy were assessed. Of the 1489 patients whose data were retrieved, 854 were included (median [range] age: 54 [18.0–83.0] years; males: 518 [60.7%]; mean [SD] Charlson Comorbidity Index score: 7.5 [3.5]). Data on second or later lines of treatment were available for 226 patients. The most common second-line therapy used after first-line steroid treatment was MMF (110 [48.67%]), followed by ibrutinib (88 [38.94%]) and ruxolitinib (28 [12.39%]). Median DoT (days) was 95 for MMF, 86 for ibrutinib, and 30 for ruxolitinib. Steroid doses were mostly kept below 0.5 mg/kg/day under all the 3 second-line treatments. These real-world data provide valuable insights into the management of cGVHD with the therapies currently used in Japan.

**Supplementary Information:**

The online version contains supplementary material available at 10.1007/s12185-026-04158-6.

## Introduction

Chronic graft-versus-host disease (cGVHD) is an immune-mediated complication resulting from histological incompatibilities and remains the primary cause of late non-relapse mortality and subsequent mortality after allogeneic hematopoietic cell transplantation (allo-HSCT).[1, 2] Approximately 30–50% of patients who undergo allo-HSCT are affected with cGVHD globally. The incidence is rising steadily due to factors such as aging, mismatched donor, peripheral blood grafts, and comorbidities.[3, 4] In Japan, the 2 year cumulative incidence of cGVHD was 35.4% with a median onset time of 4.7 months following allo-HSCT.[5] cGVHD is a heterogeneous, pleomorphic disease typically characterized by inflammation and fibrosis, affecting at least 3 organs including skin, gastrointestinal tract, mouth, liver, eyes, and lungs.[4, 6].

The treatment of cGVHD is often associated with late mortality, severe complications, and adverse events (AEs), which can reduce the quality of life (QOL) in HSCT survivors.[4] Ideally, treatment goals must include reducing symptom burden and mortality, preventing progression and disability, and improving QOL.[4] However, the standard treatment for cGVHD is still inconclusive, and clear guidelines are lacking.[7].

Immunosuppressive therapies are the cornerstone treatment for cGVHD and typically require a prolonged duration of treatment (DoT; median: 2.0–3.5 years).[4, 8] Systemic steroids (0.5–1 mg/kg/d) are used as first-line treatment; however, nearly 50% of patients require second-line therapy within 2 years.[4, 6, 8, 9] In Japan, novel treatments such as ibrutinib, ruxolitinib, and belumosudil are approved for steroid-refractory cGVHD.[10] Although ibrutinib and ruxolitinib are widely used, there is a lack of direct comparative studies between these drugs for treating steroid-refractory cGVHD. Several other second-line agents such as mycophenolate mofetil (MMF), extracorporeal photopheresis, imatinib, pentostatin, sirolimus, and interleukin-2 (IL-2) have been investigated in retrospective and prospective studies, but they have shown variable response rates and toxicities.[7] This reinforces the need for patients to continue steroids despite short- and long-term AEs such as infection, muscle weakness, bone loss, avascular necrosis, cataracts, mood changes, and hyperglycemia.[4, 7] Additionally, limited clinical data on second-line treatments for patients with steroid-refractory cGVHD can lead to wide variation for treatment regime in clinical practice.[11] According to a 2018 survey conducted by the Japan Society for Hematopoietic Cell Transplantation (JSHCT), significant variation exists in physicians’ treatment practices for cGVHD management. This highlights the need for cooperation and efforts to increase the availability of approved therapeutic agents, including the novel treatment approaches.[12] Therefore, analyzing the treatment patterns from primary to salvage therapies, including novel treatment options, as well as outcomes such as prednisone tapering, could be useful informing treatment decisions and improving patient outcomes.

## Patient and methods

### Study design and data source

A nationwide, retrospective, longitudinal, observational cohort study was conducted in patients with cGVHD using the Japan claims data from the Medical Data Vision Co., Ltd. (MDV, Tokyo, Japan) from January 01, 2003, to November 30, 2023. The MDV database is a national, patient-level, anonymized, and longitudinal claims database containing administrative claims data from over 480 hospitals, which comprises 48.27 million (as of August 2024) patients’ data since 2003. This database covers hospitalization data, hospital claims, outpatient and prescription data, and discharge summary from approximately 28% of Diagnosis Procedure Combination (DPC) hospitals (whose performance is higher than the lowest in the university hospital group) that assist to analyze data in patients with rare diseases or diseases in elderly patients.[13, 14] The MDV database encodes the disease names as per the International Classification of Diseases Version 10 (ICD-10) and Japanese Disease Name Codes. The medical procedures as per Japanese Procedure Codes.

**Fig. 1:**
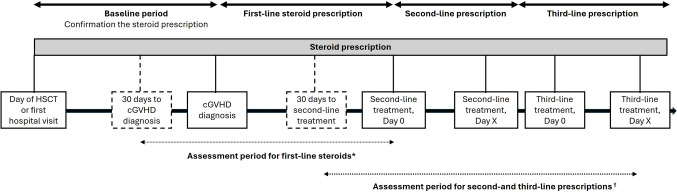
**Study design of the patients with cGVHD using MDV database.** *Due to limited number of patients with available data, the assessment period for fist-line steroids was determined to start from 30 days before cGVHD diagnosis. †Extracted ibrutinib, ruxolitinib, and mycophenolate mofetil as second- and third-line treatment. cGVHD, chronic graft-versus-host disease; MDV, medical data vision

The study consists of 4 phases: baseline, first-line steroid treatment, second-line, and third-line drug treatment periods. Baseline period was the duration between HSCT conduction day, or first hospital visit and confirmed cGVHD diagnosis and used to confirm the steroid prescription. Due to a limited number of patients with available data, assessment period for first-line steroid was started 30 days prior to diagnosis. Second or later lines of treatments included ibrutinib, ruxolitinib, and MMF (Fig. 1). As all the included data were deidentified and the patient data collected anonymously using techniques compliant with the Health Insurance Portability and Accountability Act of 1996, approval of informed consent and Institutional Review Board (IRB) approval was not required.

### Study population

**Fig. 2:**
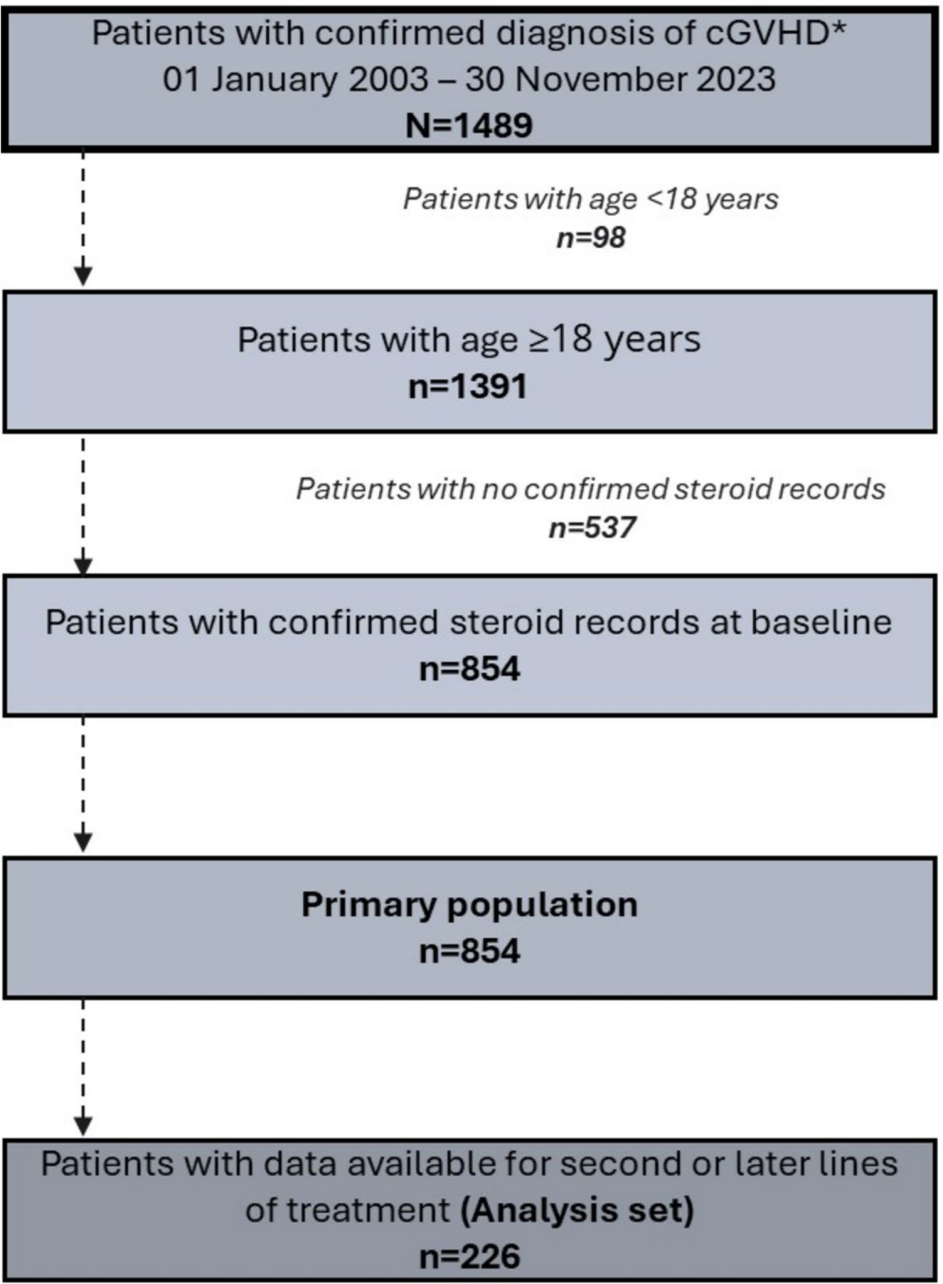
**Patient flow. **cGVHD, chronic graft-versus-host disease

Adults aged ≥ 18 years at index date with a confirmed diagnosis record of cGVHD between 2003 and 2023 were included. Index date was defined as the first record of the confirmed diagnosis of cGVHD between January 2003 and November 2023. Eligible patients had confirmed steroid prescription record at baseline (Fig. 2). Patients who were diagnosed with cGVHD on their first hospital visit record in the database (i.e., no baseline) and patients aged < 18 years were excluded.

### Study outcomes

Baseline demographics and clinical characteristics of all eligible patients (overall patients) and patients who had second or later lines of treatment records for ibrutinib, ruxolitinib, and MMF (eligible patients) were retrieved during the baseline period. DoT by line of treatment was analyzed for eligible patients and categorized as per the second or later lines of treatments. Dose reduction of prednisolone-equivalent steroids under the second-line treatment by days from diagnosis was accessed from 30 days before the start of second-line treatment (Day 30) till Day 500 for ibrutinib, ruxolitinib, and MMF in eligible patients.

### Statistical analysis

Baseline demographics and clinical characteristics, DoT, and dose reduction of prednisolone-equivalent steroids were calculated using descriptive statistics and presented as mean (SD), median (min–max), n, and percentages. DoT in second-line parameters was evaluated using Kaplan–Meier analysis. Dose reduction of prednisolone-equivalent steroids under the second-line treatment from the first-treatment, from Day 30 to Day 500, was also assessed using a paired t-test and presented as a box plot. All analyses were performed using statistical analysis software (SAS) Version 9.4 (SAS Institute Inc. Cary, NC, USA) and R version 4.2.0.

## Results

### Baseline demographic and clinical characteristics

From the MDV database, a total of 1489 patients with cGVHD requiring systemic steroid therapy were retrieved. Of which, 854 patients were included after screening, and formed the overall population. Of these, 226 patients had the record of second or later lines of treatment for cGVHD and formed the analysis population (Fig. 2). The median age (range) was 54 (18.0–83.0) years, with a predominance of males (*n* = 518, 60.7%; Table 1). Overall, 704 (82.4%) patients were aged 18–64 years, and 617 (72.2%) had index data between 2017 and 2023. The mean (SD) duration from index date to start of non-steroid treatment was 2.5 (8.5) months. The mean (SD) CCI score was 7.5 (3.5), and 821 (96.1) patients had a high CCI. Most common baseline comorbidities were malignancies (including lymphoma and leukemia [*n* = 813, 95.2%]), mild liver disease (*n* = 648, 75.9%), congestive heart failure (*n* = 614, 71.9%), and chronic pulmonary disease (*n* = 577, 67.6%). The baseline demographic and clinical characteristics of the analysis population with 2L or later treatment line (*n* = 226) were similar to the overall population (Supplementary Table 1).

**Table 1:** Baseline demographics and clinical characteristics in patients with cGVHD

Characteristics	*N* = 854
*Age at index, years*	
Mean (SD)	51.2 (13.9)
Median (min–max)	54 (18.0–83.0)
*Age group at index, years, n (%)*	
18–64	748 (83.2)
≥ 65	151 (16.8)
*Gender, n (%)*	
Male	518 (60.7)
Female	336 (39.3)
*Year of the index date group, n (%)*	
2010–2016	237 (27.8)
2017–2023	617 (72.2)
*Year of start of treatment, n (%)*	
2003–2009	2 (0.2)
2010–2016	380 (44.5)
2017–2023	472 (55.3)
Time from first SCT to index date, months, mean (SD)	16.1 (16.7)
Time from index date to start of non-steroid therapy, months, mean (SD)	2.5 (8.5)
*CCI score*	
Mean (SD)	7.5 (3.5)
Median (min–max)	7.0 (0.0–17.0)
*CCI score group, n (%)*	
0	4 (0.5%)
1	15 (1.8%)
2	14 (1.6%)
3	43 (5.0%)
≥ 4	778 (91.1%)
Comorbidities*	
Any malignancy including lymphoma and leukemia^†^	813 (95.2)
Mild liver disease	648 (75.9)
Congestive heart failure	614 (71.9)
Chronic pulmonary disease	577 (67.6)
Peptic ulcer disease	514 (60.2)
Rheumatic disease	351 (41.1)
Renal disease	256 (30.0)
Diabetes without chronic complications	213 (24.9)
Metastatic solid tumor	194 (22.7)
Cerebrovascular disease	191 (22.4)

CCI, Charlson Comorbidity Index; cGVHD, chronic graft-versus-host disease; SD, standard deviation

^*^Data presented for > 5% patients; ^†^except malignant neoplasm of skin

### Duration of treatment

**Fig. 3:**
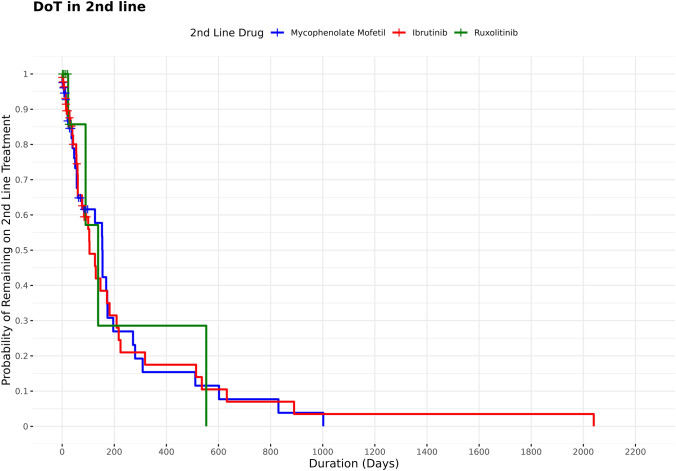
Duration of treatment for second-line treatment regimen in patients with cGVHD. cGVHD, chronic graft-versus-host disease.

Overall, 226 patients used second-line, 72 used third-line, 39 used fourth-line, 24 used fifth-line, and 13 used sixth-line therapies for cGVHD. After the first-line steroids, the most frequent second-line treatment was MMF (*n* = 110, 48.67%), ibrutinib (n = 88, 38.94%), and ruxolitinib which were added on to the steroid treatment (*n* = 28, 12.39%) in patients with cGVHD (Table 2). The DoT (mean [SD]) for second-line therapy for MMF, ibrutinib, and ruxolitinib was 191.20 (270.16), 135.10 (134.27), and 46.80 (57.31) days, respectively, whereas the median DoT for MMF, ibrutinib, and ruxolitinib was 95 (4–1597), 86 (3–665), and 30 (3–225) days, respectively (Supplementary Table 1, Fig. 3). Among these 3 regimens, most of the patients used MMF as third-line (*n* = 36, 50.0%) and fourth-line (*n* = 21, 53.80%) agent with a mean (SD) DoT of 111.10 (212.56) days in third line and 64.80 (76.20) days in the fourth line. In the third line, 31 (43.10%) patients used ibrutinib with mean (SD) DoT of 77.91 (112.52) days, and 5 (6.90%) used ruxolitinib with mean (SD) DoT of 12.6 (10.26) days. The median DoT from third-line therapy was 36 (2–1149) days for MMF, 32 (2–449) days for ibrutinib, and 9 (2–29) days for ruxolitinib (Table 2). Overall, 21 (53.80%), 17 (43.60%), and 1 (2.60%) patient used MMF, ibrutinib and ruxolitinib as fourth-line agent, respectively, with mean (SD) DoT of 64.80 (76.20) days, 71.10 (130.30) days, and 8 (0) days, respectively. Only MMF was used as a sixth (*n* = 13) line agent (Table 2).

**Table 2:** **Duration of therapy for treatment regimen in patients with cGVHD**

Line of therapy	Treatments	*n* (%)	DoT, days	
			Mean (SD)	Median (Range)
Second line (*N* = 226)	MMF	110 (48.67%)	191.20 (270.16)	95 (4–1597)
	Ibrutinib	88 (38.94%)	135.10 (134.27)	86 (3–665)
	Ruxolitinib	28 (12.39%)	46.80 (57.31)	30 (3–225)
Third line (*N* = 72)	MMF	36 (50%)	111.10 (212.56)	36 (2–1149)
	Ibrutinib	31 (43.10%)	77.91 (112.52)	32 (2–449)
	Ruxolitinib	5 (6.90%)	12.6 (10.26)	9 (2–29)
Fourth line (*N* = 39)	MMF	21 (53.80%)	64.80 (76.20)	29 (2–252)
	Ibrutinib	17 (43.60%)	71.10 (130.30)	33 (3–564)
	Ruxolitinib	1 (2.60%)	8 (0)	8 (8–8)
Fifth line (*N* = 24)	MMF	16 (66.7%)	96.30 (119.15)	18.5 (3–335)
	Ibrutinib	8 (33.33%)	24.10 (24.66)	13.5 (3–62)
Sixth line (*N* = 13)	MMF	13 (100%)	47.90 (54.32)	29 (2–187)

cGVHD, chronic graft-versus-host disease; DoT, duration of therapy; MMF, mycophenolate mofetil

### Change in daily steroid dose

**Fig. 4:**
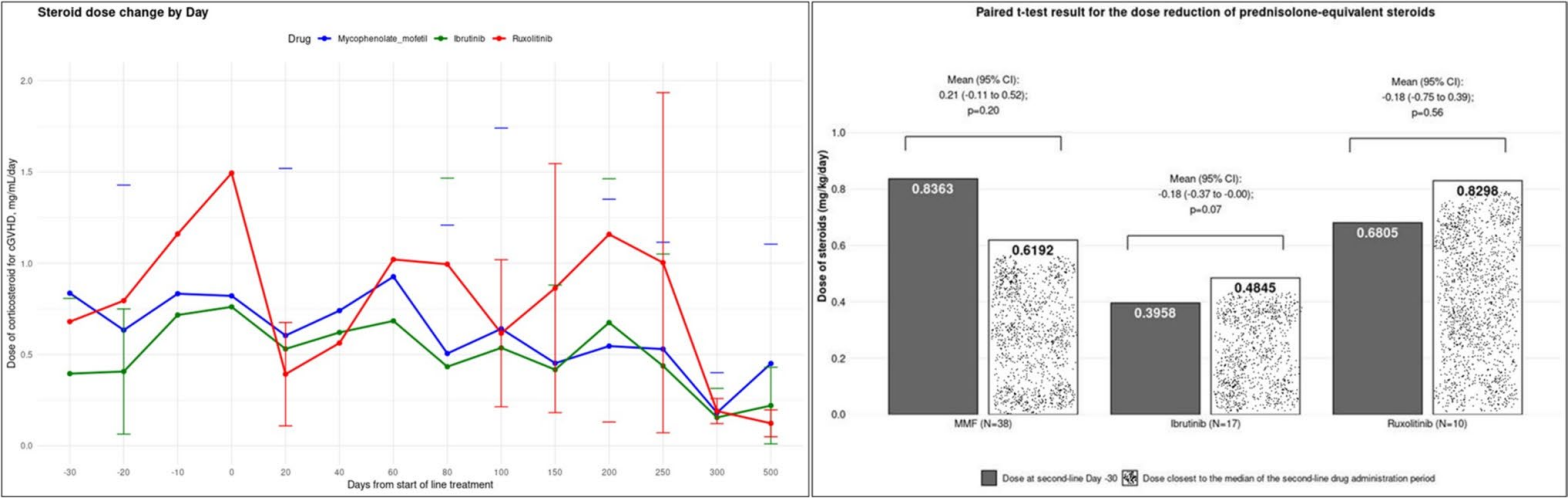
Dose reduction of prednisolone-equivalent steroids. **A** The dose reduction of prednisolone-equivalent steroids under the second-line treatment by days from the diagnosis. **B** Paired t-test result for the dose reduction of prednisolone-equivalent steroids under the second-line treatment from the first line. cGVHD, chronic graft-versus-host disease; CI, confidence interval; MMF, mycophenolate mofetil; SD, standard deviation

The dose reduction of prednisolone-equivalent steroids was assessed from Day-30 till Day 500 (Fig. 4A). At each time point (except Day 250 and Day 500 for ruxolitinib), the median doses of steroids were mostly managed below 0.5 mg/kg/day for all 3 second-line treatments. After initiation of second-line treatments (from Day 0 to Day 500), the median doses of prednisolone-equivalent steroids were 0.10–0.44 mg/kg/day for MMF group, 0.14–0.33 mg/kg/day for ibrutinib group, and 0.12–0.69 mg/kg/day for ruxolitinib group (Supplementary Table S2). For the ibrutinib group, the mean and median steroid doses were < 0.5 mg/kg/day, irrespective of the time points. On Day 0, the mean steroid dose was 0.83 mg/kg/day for the MMF group, 0.76 mg/kg/day for ibrutinib group, and 1.49 mg/kg/day for ruxolitinib group. For patients on MMF, the mean and median steroid doses were below 1.0 mg/kg/day by Day 20 and dropped to below 0.5 mg/kg/day from Day 150 onward. In the ruxolitinib group, the mean and median doses were below 1.0 mg/kg/day after Day 100, and median doses were generally below 0.5 mg/kg/day except between Day 60 and Day 100. The closest time point to the median of each DoT was Day 100 for MMF and ibrutinib, and Day 40 for ruxolitinib, after initiation of second-line treatments. The reduction in prednisolone-equivalent steroid dose from first-line to second-line treatment was not statistically significant. The mean difference (mg/kg/day) in prednisolone-equivalent steroids dose between 30 days before start of second-line and time closest to start of second-line treatment was 0.21 (−0.11 to 0.52, *p* = 0.20) for MMF, −0.18 (−0.37 to 0.07, *p* = 0.07) for ibrutinib, and −0.18 (−0.75 to 0.39, *p* = 0.56) for ruxolitinib (Fig. 4B).

## Discussion

This retrospective observational study offers a comprehensive overview on the treatment pattern and clinical outcomes in Japanese patients with cGVHD in a real-world setting. This study highlights the DoT, TTNT, and the dose reduction with first-line steroids after initiation of second or later lines of therapy in patients with cGVHD using claims data from the largest database in Japan. Our analysis found that MMF was the most frequently used second or later line of drug followed by ibrutinib and ruxolitinib, respectively. With the use of these agents, the prednisolone-equivalent corticosteroid doses were mostly maintained below 0.5 mg throughout the study period.

The baseline demographics and clinical characteristics of this analysis are consistent with those reported in the literature on cGVHD outcomes.[15–20] The median age was reported > 50 years, [16, 17, 19, 21] and most of the patients had cGVHD below 60 years of age.[18] However, there are mixed data on gender specific prevalences of cGVHD.[15–21] Patients with cGVHD are always associated with higher comorbidities than pre-HSCT, [22] and a retrospective analysis using Komodo closed claims database reported a mean CCI score of 3.6 in patients with cGVHD.[21] In contrast, our analysis found the mean CCI score of 7.5 and > 90% patients with CCI score ≥ 4, highlighting the severity of patients with cGVHD in Japan compared to global population, and need for healthcare attention to these specific population.

In this analysis, patients were assessed for the treatment pattern 30 days prior to the cGVHD diagnosis. In consistent with these findings, a retrospective study found that approximately 68% of patients initiated the systemic therapy 30 days prior to cGVHD diagnosis; and the rates were higher > 80% prior to 6 months of cGVHD diagnosis. This might be due to some patients already on treatment for acute GVHD before proceeding to cGVHD.[16].

In our analysis, more than 45% of patients used MMF as a second or later line of therapy. Approximately 40% of patients received ibrutinib as second-line treatment, nearly 45% as third- or fourth-line therapy, and more than 30% as fifth-line therapy. Ruxolitinib was used in over 10% of patients as a second-line agent, around 7% as third-line, and approximately 3% as fourth-line therapy. MMF has been approved in patients with GVHD and is widely used as combination therapy for treatment of cGVHD, but not added to the first-line treatment.[6, 23] Prior to MMF receiving approval for GVHD indication, a retrospective study using patients’ data (*N* = 39,941) from Japanese Data Center for Hematopoietic Cell Transplantation (JDCHCT) reported that MMF was the most frequently used drug for Japanese patients with GVHD as it was readily available in Japanese market for other indications (renal transplantation, solid organ transplant, lupus nephritis).[24].

In terms of treatment duration, median DoT was numerically higher for MMF, followed by ibrutinib and ruxolitinib during second line of treatment. However, a retrospective study suggested that ruxolitinib and extracorporeal photopheresis treatment had the highest DoT during the second-line treatment in patients with cGVHD.[16] MMF had a numerically longer DoT during third or later lines of therapy among the 3 agents, except ibrutinib was numerically longer in fourth line. Furthermore, in this analysis, DoT decreased as the number of therapy lines increased, which is consistent with published literature.[16] This implies rapid treatment cycling in patients with cGVHD, highlighting the need for more effective treatment options for the management of cGVHD.

The median DoT was 95 (4–1597) days for MMF as the second line, while for ibrutinib, the median DoT in fourth line of therapy was 33 (3–564) days. The median duration of second, third, and fourth line of therapy was 81–101 days, 84–106 days, and 10–108 days. Consistent with these findings, a retrospective study (*N* = 5259) from US using data from Medicare Fee-for-Service and PharMetrics reported that HSCT recipients were on second third, and fourth lines of treatment for 121, 114, and 106 days, respectively.[16].

As long-term steroids are associated with treatment-related complications, clinicians used to taper steroids in patients with cGVHD considering the benefit-risk approach. Rapid tapering increases the risk of cGVHD flare and non-relapse mortality while slow tapering increases the risk of steroid-related toxicities.[25] Here, the median dose of prednisolone-equivalent steroids was reduced 0.5 mg/kg/day in most of times from Day 0 to Day 500 under all 3 second-line agents (MMF, ibrutinib, ruxolitinib). Patients treated with MMF (0.1–0.4 mg/kg/day) and ibrutinib (0.1–0.3 mg/kg/day) were managed at a lower steroid dose than those treated with ruxolitinib (0.3–1.0 mg/kg/day). These findings are in line with published literature highlighting the steroid sparing effect with these agents when used during second or later lines therapy. A retrospective study (*N* = 34) from Hope National Medical Center reported that 14 out of 22 patients who used MMF as salvage therapy had a median of 50% (range: 25–100%) reduction is steroid (prednisolone) dose in a minimum of 6-month observation period. The baseline steroid dose was reduced from 5–120 mg/day to 0–40 mg/day over 6 month period.[26] An open-label study reported a reduction in median corticosteroid dose among responders from 0.29 mg/kg per day at baseline to 0.12 mg/kg per day at week 49, in patients who failed ≥ 1 therapy for cGVHD.[27, 28] Additionally, a phase 1b/2 study reported a reduction in steroid dose from 0.3 to 0.1 mg/kg/day at week 52 in 64% of responders after ibrutinib therapy.[29] Similarly, a retrospective single-center observational study *N* = 48) from Spain found that after a median follow-up of 11 months, ruxolitinib reduced steroid doses from 0.30 to 0.13 mg/kg, with 50% of patients achieving a 50% reduction.[30] The interim analysis of single-arm, multicenter, phase 2 REACH5 study also reported similar results.[31] As steroid tapering is considered as important endpoint in patients with steroid-resistant cGVHD, our analysis could provide insights for clinicians in developing suitable treatment therapies based on the benefit-risk approach.

As this retrospective study was conducted in a country-specific population, findings should be interpreted with caution in the general population. Although MDV is a large claim database in Japan, it only includes data from insured or hospitalized patients, and not patients from outside hospitals or who switch hospitals. In addition, the diagnostic criteria for cGVHD have evolved over time (with the NIH 2014 criteria now widely adopted), and it was not possible to determine which specific criteria were applied for each patient, potentially introducing heterogeneity in case identification, but all included patients had confirmed diagnosis codes consistent with chronic GVHD in the claims database. This analysis did not analyze the treatment patterns in patients with cGVHD as per the disease severity. Some severe patients might have received high dose steroids or immunosuppressive agents that were not recorded. All other limitations of a real-world study also apply to this analysis. Despite the limitations, our analysis covers the largest patient population in Japanand could provide insight on recent treatment patterns for patients with cGVHD in a real-world setting.

Currently, management of cGVHD is a rising concern in healthcare system as the incidence grows due to increased number of alloy-HSCT survivors and improvements in transplantation procedure.[32] Moreover, challenged increase in management of steroid-refractory patients as second-line therapies provides variable response with no definite comparative data available between ibrutinib and ruxolitinib (mostly used agents). This study provides insight into the current treatment pattern and steroid usage in patients with cGVHD which could be useful for the clinicians to inform treatment decision making.

## Conclusions

The current analysis evaluated the treatment pattern and clinical outcomes in patients with cGVHD from Japan using MDV database, which provided real-world illustrations of currently used therapies, DoT for MMF, ibrutinib, and ruxolitinib when used during second or later line of therapy, along with prednisolone-equivalent steroid dose reductions under second-line therapy. The treatment regimen of MMF as second-line therapy had the highest median DoT numerically, followed by ibrutinib. However, ibrutinib showed a numerically longer DoT in fourth line compared to MMF. Steroids were mostly managed below 0.5 mg/kg/day across all 3 second-line treatments. Thus, this study provides valuable clinical insights into the therapies used in Japanese patients with cGVHD. Further investigations must explore the treatment patterns and clinical outcomes including more therapeutic agents used for cGVHD, and in patients with different disease severities.

## Supplementary Information

Below is the link to the electronic supplementary material.

Supplementary file1 (DOCX 142 KB)

## Data Availability

The data that support the findings of this study are available from the MDV database. Restrictions apply to the availability of these data, which were used under license for this study.
