# Supplementary material for: Evaluation of Real-world treatment patterns in Japanese patients with cGVHD: A retrospective claims database study
Source: Int J Hematol. 2026 Mar 18;123(6):896–904. doi: 10.1007/s12185-026-04158-6 (PMC13233629; doi:10.1007/s12185-026-04158-6)

### **Supplementary Table 1. Baseline demographics and clinical characteristics in patients with cGVHD with 2L or later treatment line**

| Characteristics | N=226 |
| --- | --- |
| Age at index, years | |
| Mean (SD) | 48.8 (13.9) |
| Median (min-max) | 50 (18.0-73.0) |
| Age group at index, years, n (%) | |
| 18-64 | 195 (86.3) |
| ≥65 | 31 (13.7) |
| Gender, n (%) | |
| Male | 146 (64.6) |
| Female | 80 (35.4) |
| Year of the index date group, n (%) | |
| 2010-2016 | 33 (14.6) |
| 2017-2023 | 193 (85.4) |
| Year of start of treatment, n (%) | |
| 2010-2016 | 68 (30.1) |
| 2017-2023 | 158 (69.9) |
| Time from first SCT to index date, months, mean (SD) | 12.2 (11.8) |
| Time from index date to start of non-steroid therapy, months, mean (SD) | 2.2 (7.6) |
| CCI score | |
| Mean (SD) | 8.4 (3.5) |
| Median (min-max) | 8.0 (1.0-17.0) |
| CCI score group, n (%) | |
| 1 | 1 (0.4%) |
| 2 | 3 (1.3%) |
| 3 | 8 (3.5%) |
| ≥4 | 214 (94.7%) |
| Comorbidities* | |
| Any malignancy including lymphoma and leukemia^†^ | 220 (97.3) |
| Mild liver disease | 183 (80.9) |
| Congestive heart failure | 177 (78.3) |
| Chronic pulmonary disease | 173 (76.5) |
| Peptic ulcer disease | 149 (65.9) |
| Rheumatic disease | 112 (49.5) |
| Renal disease | 91 (40.3) |
| Diabetes without chronic complications | 65 (28.8) |
| Metastatic solid tumor | 58 (25.7) |
| Cerebrovascular disease | 68 (30.1) |

*Data presented for >5% patients; ^†^except malignant neoplasm of skin

CCI, Charlson Comorbidity Index; cGVHD, chronic graft versus host disease; SD, standard deviation.

### **Supplementary table S1: Summary of the dose reduction of prednisolone-equivalent steroids under the second-line treatment by days from the diagnosis (mg/kg/day)**

| Drugs | MMF | | | Ibrutinib | | | Ruxolitinib | | |
| --- | --- | --- | --- | --- | --- | --- | --- | --- | --- |
|  | N | Mean (SD) | Median  (min-max) | N | Mean (SD) | Median  (min-max) | N | Mean (SD) | Median (min-max) |
| Day -30 | 80 | 0.84 (1.26) | 0.51 (0.00–11.88) | 77 | 0.40 (0.41) | 0.28 (0.00–2.90) | 23 | 0.68 (1.46) | 0.27 (0.00–9.31) |
| Day -20 | 87 | 0.63 (0.79) | 0.46 (0.00–11.88) | 85 | 0.41 (0.34) | 0.33 (0.00–2.81) | 25 | 0.80 (1.79) | 0.30 (0.02–10.89) |
| Day -10 | 94 | 0.83 (1.90) | 0.45 (0.00–20.23) | 81 | 0.72 (1.76) | 0.33 (0.00–16.89) | 28 | 1.16 (2.56) | 0.37 (0.00–16.89) |
| Day 0 | 101 | 0.83 (2.12) | 0.44 (0.00–27.23) | 87 | 0.76 (2.24) | 0.33 (0.00–27.23) | 28 | 1.49 (4.25) | 0.37 (0.01–31.25) |
| Day 20 | 93 | 0.61 (0.91) | 0.36 (0.00–9.69) | 83 | 0.55 (1.88) | 0.27 (0.00–28.60) | 24 | 0.39 (0.28) | 0.44 (0.00–1.44) |
| Day 40 | 84 | 0.74 (1.64) | 0.28 (0.00–19.38) | 78 | 0.67 (2.01) | 0.35 (0.01–22.44) | 22 | 0.56 (2.27) | 0.30 (0.00–22.44) |
| Day 60 | 72 | 0.93 (2.30) | 0.33 (0.00–19.38) | 70 | 0.73 (2.30) | 0.27 (0.00–19.38) | 16 | 1.02 (2.49) | 0.48 (0.04–16.89) |
| Day 80 | 70 | 0.51 (0.70) | 0.22 (0.00–2.79) | 62 | 0.51 (1.15) | 0.20 (0.00–16.89) | 18 | 0.99 (1.83) | 0.48 (0.00–16.89) |
| Day 100 | 64 | 0.64 (1.10) | 0.29 (0.01–12.78) | 58 | 0.62 (2.12) | 0.20 (0.00–19.03) | 16 | 0.62 (0.40) | 0.69 (0.00–2.00) |
| Day 150 | 48 | 0.46 (1.58) | 0.17 (0.00–15.51) | 47 | 0.48 (0.64) | 0.21 (0.00–4.23) | 13 | 0.86 (0.68) | 0.43 (0.06–1.70) |
| Day 200 | 38 | 0.55 (0.80) | 0.17 (0.00–4.00) | 33 | 0.72 (0.87) | 0.32 (0.03–4.23) | 12 | 1.16 (1.03) | 1.49 (0.03–4.00) |
| Day 250 | 33 | 0.53 (0.58) | 0.33 (0.00–2.00) | 29 | 0.45 (0.61) | 0.20 (0.03–2.00) | 9 | 1.00 (0.93) | 0.30 (0.03–2.00) |
| Day 300 | 23 | 0.18 (0.22) | 0.10 (0.00–1.21) | 24 | 0.16 (0.16) | 0.14 (0.02–1.21) | 6 | 0.19 (0.07) | 0.24 (0.05–0.25) |
| Day 500 | 16 | 0.48 (0.66) | 0.10 (0.00–1.98) | 12 | 0.27 (0.27) | 0.25 (0.03–1.33) | 5 | 0.12 (0.07) | 0.12 (0.07–0.30) |

MMF, Mycophenolate mofetil; SD, standard deviation

### **Supplementary Figure 1. Sankey diagram depicting transitions in systemic therapies among patients with cGVHD requiring systemic steroid therapy.**


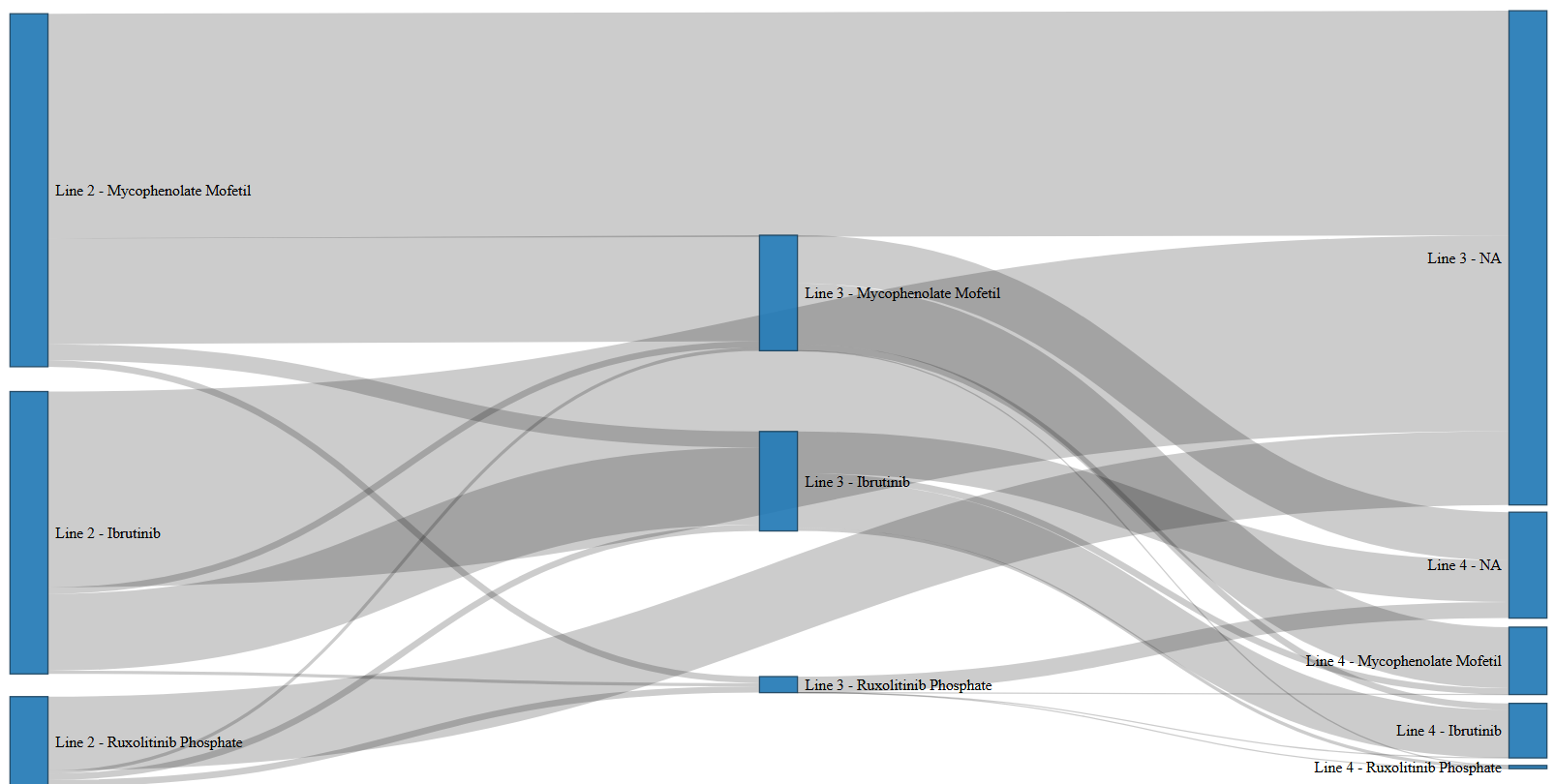

Supplement: Supplementary file 1 — Supplementary file1 (DOCX 142 KB) [file 12185_2026_4158_MOESM1_ESM.docx]
